# Improved Application of Carbon Nanotube Atomic Force Microscopy Probes Using PeakForce Tapping Mode

**DOI:** 10.3390/nano8100807

**Published:** 2018-10-09

**Authors:** Ashley D. Slattery, Cameron J. Shearer, Joseph G. Shapter, Adam J. Blanch, Jamie S. Quinton, Christopher T. Gibson

**Affiliations:** 1 Flinders Institute for NanoScale Science and Technology, College of Science and Engineering, Flinders University, Bedford Park, SA 5042, Australia; Ashley.slattery@adelaide.edu.au (A.D.S.); cameron.shearer@adelaide.edu.au (C.J.S.); j.shapter@uq.edu.au (J.G.S.); adam.blanch@unimelb.edu.au (A.J.B.); 2 Adelaide Microscopy, The University of Adelaide, Adelaide, SA 5005, Australia; 3 Department of Chemistry, The University of Adelaide, Adelaide, SA 5005, Australia; 4 Australian Institute for Bioengineering and Nanotechnology, The University of Queensland, Brisbane, QLD 4072, Australia; 5 Department of Biochemistry and Molecular Biology, Bio21 Institute, University of Melbourne, Melbourne, VIC 3010, Australia

**Keywords:** carbon nanotubes, atomic force microscope tips, tapping mode, PeakForce tapping mode, imaging artefacts

## Abstract

In this work PeakForce tapping (PFT) imaging was demonstrated with carbon nanotube atomic force microscopy (CNT-AFM) probes; this imaging mode shows great promise for providing simple, stable imaging with CNT-AFM probes, which can be difficult to apply. The PFT mode is used with CNT-AFM probes to demonstrate high resolution imaging on samples with features in the nanometre range, including a Nioprobe calibration sample and gold nanoparticles on silicon, in order to demonstrate the modes imaging effectiveness, and to also aid in determining the diameter of very thin CNT-AFM probes. In addition to stable operation, the PFT mode is shown to eliminate “ringing” artefacts that often affect CNT-AFM probes in tapping mode near steep vertical step edges. This will allow for the characterization of high aspect ratio structures using CNT-AFM probes, an exercise which has previously been challenging with the standard tapping mode.

## 1. Introduction

Carbon nanotube atomic force microscopy (CNT-AFM) probes have demonstrated that they can provide high-resolution imaging of a number of different types of samples, including biomolecules [1,2,3], nanomaterials [4,5,6], and polymers [7]. Recently, Shearer et al. demonstrated that CNT-AFM probes can be particularly effective at determining the thickness of 2D nanomaterials such as graphene [6], and Slattery et al. [8] also recently showed that CNT-AFM probes can achieve high resolution conductivity imaging of surfaces. There are a number of methods for CNT attachment to AFM tips which include direct CNT growth on the AFM tip by chemical vapor deposition (CVD) [9,10], manual attachment using optical or electron microscopy [11,12], manual attachment through imaging CNT covered surfaces [4,5,13] and dielectrophoresis [14,15,16]. Some methods use multi-walled CNTs (MWCNT) as the CNT source, while others use single-walled CNTs (SWCNT). While an AFM probe with an individual SWCNT attached to the tip should, on average, yield CNT-AFM probes with smaller diameters, MWCNT probes should provide greater stability due to their increased diameters. This will depend somewhat on the aspect ratio (length to width) of the attached CNT, regardless of whether it is SWCNT or MWCNT. It should also be noted that CNT-AFM probes can also employ bundles of SWCNTs at the very end, which can result in diameters from 5 nm to 50 nm [8,16], and therefore, they could have diameters that are similar to multi-walled nanotubes. The method of CNT attachment described in this work results in very thin SWCNT bundles or fibers attached to the AFM tips. The wear-resistant properties are expected to be similar for both types of CNT-AFM probes.

The application of CNT-AFM probes has unfortunately been hindered due to a number of factors, including the degree of adherence of the CNT to the AFM tip, and also their varied and unstable behavior compared to that of standard silicon probes. While CNTs are extremely rigid in their axial direction, they are quite flexible in the lateral direction and they buckle easily, which can produce complications for stable imaging. There have been a number of papers investigating the dynamics of CNT-AFM probes during imaging, highlighting the challenge posed by using these specialized probes [17,18]. Improved understanding of CNT–sample interactions will assist researchers to better apply these probes, however they still remain difficult to fabricate and use.

### 1.1. Imaging with CNT-Modified AFM Probes

There are a number of important factors that must be controlled effectively when AFM imaging with a CNT-AFM probe, to ensure that the CNT remains stable, but perhaps the most critical is the applied force. Excessive force can cause the nanotube to buckle, resulting in an unstable interaction [19,20]. CNT buckling results in unstable imaging, often causing imaging artefacts, and if the force applied is significant or if the CNT is weakly attached, then the CNT may break free from the tip. Buckling poses a significant problem if the CNT is too long or if it is not perpendicular to the surface. The orientation and length of the CNT are critical factors in probe fabrication for these reasons, making the fabrication requirements of these probes highly demanding. For the reasons described above, CNT-AFM probes are not well suited to imaging in contact mode, as the high lateral forces buckle the nanotube easily. In order to reduce lateral forces, CNT-AFM probes are most commonly applied in tapping mode.

### 1.2. Tapping Mode

**Figure 1:**
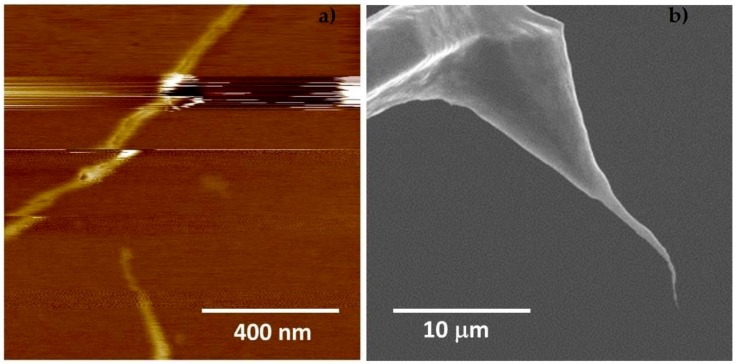
(**a**) An atomic force microscopy(AFM) image of carbon nanotubes on silicon obtained in tapping mode with a long carbon nanotube (CNT) (~10 µm) attached to a tapping mode probe. (**b**) shows the corresponding scanning electron microscope (SEM) image for the probe used to acquire the AFM image in Figure 1a. The instability in the AFM image is clear. The height or color scale of the AFM image is 20 nm.

Tapping mode is widely used in AFM systems, primarily due to its ability to image samples with reduced lateral forces and thus reduced tip wear. For most applications, the tapping amplitude is tens of nanometres, and it must be large enough to prevent tip-surface adhesion due to meniscus or capillary forces. CNT-AFM probes are often applied using tapping mode, and they require careful operation with very small amplitudes, to reduce the imaging force and the possibility of destabilizing the nanotube. While tapping mode is generally a robust imaging mode and it is still used extensively, it has several inherent disadvantages. Operation at cantilever resonance produces a complicated interaction that can make imaging difficult, and imaging forces vary with sample properties such as roughness and modulus, which can cause tip damage. The imaging set-point force represents an average over the entire oscillation trajectory and a number of oscillation cycles. This makes it difficult to quantify and extract material properties, and it also reduces the sensitivity to the high-resolution forces at small tip-sample separation. Figure 1a shows a tapping mode AFM image of carbon nanotubes on silicon using a relatively long CNT-AFM tip (the CNT is ~10 µm long), as can be seen in Figure 1b, and the instability produced in the AFM image is evident. The AFM-CNT probe used to generate the data in Figure 1 was the same as that studied in Figure 8 of Slattery et al. [16]. A rigid silicon tip by contrast, exhibits rapid, monotonic damping of the cantilever amplitude as the tip-sample distance decreases, which provides stable imaging feedback.

Another disadvantage of tapping mode imaging is that cantilevers with relatively high resonant frequencies are required, and hence the spring constant of these cantilevers is also quite high. If the cantilever is as soft as possible, then (in static operation) more deflection of the cantilever will occur before the CNT begins to buckle. Due to their extremely high aspect ratios, CNT probes have also been proposed as excellent candidates for the measurement of trenches with steep, vertical sidewalls. These features require accurate measurement for quality control in the semiconductor industry. This application has been hindered by the presence of the “ringing” artefact first reported by Park et al., which occurs when a CNT probe is in close proximity with a steep sidewall [20,21]. Strus et al. also investigated this ringing artefact in detail [22]. This is discussed in more detail in Section 3.1.

### 1.3. PeakForce Tapping Mode

In the last 10 to 15 years, a number of new imaging modes have emerged, which provide new and detailed information about surfaces and their adhesive and mechanical properties. Some examples include topography and recognition imaging (TREC) [23,24,25] and Harmonix imaging [26,27]. PeakForce tapping (PFT) mode is also another relatively new imaging mode offered by Bruker on many of their AFM instruments, and it has been in use since 2005. Briefly summarizing, PFT is an imaging mode that involves intermittent sample surface contact up to a set applied force, with the sample surface oscillating relative to the AFM tip at a frequency of 0.5–8 kHz at an amplitude that is set by the user. This mode of operation allows the user to simultaneously measure a number of surface properties including peak interaction force, adhesion, and topography. The peak repulsive interaction force is measured in real-time, and it is used as the feedback signal. This allows the PFT mode to reduce the effects of common image artefacts observed with other AFM techniques, such as tapping mode, which tend to be dominated by tip-sample adhesion.

It is important to note that while PFT is unique to Bruker and it is the main focus of this work, there are other manufacturers that offer similar imaging modes. JPK instruments include a quantitative imaging (QI) mode with the family of NanoWizard 3 AFMs, which operate in a similar manner to PFT. One of the key advantages of PFT is that the tip–sample interaction force is directly measured, and is used as the feedback for imaging. This allows a constant imaging force to be maintained over the entire scan, which is critical for avoiding instabilities due to CNT buckling, and also enables the imaging of forces in the pN range.

Optimisation of probe tracking in tapping mode is dependent on the dynamics of the probe, and it requires the gains and set-point to be carefully adjusted to achieve good feedback. This process is generally quite simple on basic surfaces with standard probes; however, the introduction of a complex surface or probe (i.e., the CNT-modified probe) often complicates the feedback, and thus the required gain and set-point optimization process is significantly more challenging. A substantial benefit of PFT is that the feedback interaction is greatly simplified, which makes the transition from standard silicon probes to CNT probes much easier to understand and apply. In addition to this, constant display/analysis of PFT’s real-time force curve output allows for the interaction of the CNT with the surface to be monitored for processes such as buckling, enabling subsequent correction of feedback parameters.

Another advantage of PFT is the analysis of force curves using the PeakForce-quantitative nanomechanical mapping (PF-QNM) mode, which can measure quantitative tip–sample interaction information; provided that the appropriate calibrations of the cantilever spring constant [28,29,30,31], the deflection sensitivity [32,33,34] and tip shape [35,36,37] are performed. Wear resistance of the CNT results in a stable tip radius, which is highly desirable for quantitative analysis, allowing for consistent measurement of interactions during imaging.

## 2. Materials and Methods

### 2.1. Carbon Nanotube Attachment to AFM Tips

The SWCNT attachment process has been developed in our previous work and described in detail [38]; however we will briefly summarize the process here. The method was carried out within a SEM (FEI_Helios D344_Nanolab) (Eindhoven, Netherlands) fitted with a micromanipulator. A free-standing SWCNT film, which is commonly known as ‘buckypaper’, was fabricated by vacuum filtration of arc-discharge-produced SWCNTs (0.2 mg mL^−1^, AP-SWCNT, Carbon Solutions Inc. (Riverside CA, USA) in 0.5 wt % sodium dodecylbenzenesulfonate (SDBS, Sigma-Aldrich Sydney, Australia), and this was used as the SWCNT source. A piece of buckypaper was fixed to the end of a micromanipulator needle using carbon paste, and a small section of buckypaper was then torn in the direction away from the needle in order to produce a straight edge with aligned SWCNTs, which tend to form very thin bundles or fibers. A Helios D344 Dualbeam (FEI, Eindhoven, Netherlands) instrument was used to image the attachment process and to cut the CNTs. Platinum precursor (trimethyl[(1,2,3,4,5-ETA)-1 methyl 2,4 cyclopentadien-1-YL] platinum FEI, Eindhoven, Netherlands) and a water source (magnesium sulfate heptahydrate FEI, Eindhoven, Netherlands) were injected onto the sample through needles located approximately 200 μm from the electron beam. The AFM probe was first brought into contact with a dangling SWCNT, and non-covalent forces held the CNT onto the AFM probe, then the SWCNT was cut by directing the electron beam at the CNT about 100 nm from the AFM probe with the water source injected. Finally, Pt was injected and the SWCNT and AFM tip were welded together using electron beam deposition. Most imaging, deposition, and cutting processes was performed at a beam energy of 1 keV. The micromanipulator used to position the CNTs on the AFM probes was a MM3A model (Kleindiek Reutlingen, Germany). During a typical transfer, a SWCNT-modified AFM probe was fabricated over 15–20 min, and the yield of the functional probes was approximately 80 to 90%.

### 2.2. Gold Nanoparticle Sample Preparation

The gold nanoparticles were prepared using a similar approach to that of Flavel et al. the procedure for which is briefly reported here [39]. Prior to the reaction, glassware was cleaned in a solution of 3:1 (*v*/*v*) hydrochloric acid (36%, RCI Labscan Bangkok, Thailand) and nitric acid (70%, RCI Labscan Bangkok, Thailand). A volume of 1 mL of 1% aqueous gold (III) chloride solution (99.9985%, Proscitech Kirwan, Queensland) was then added to 100 mL water under stirring and allowed to disperse for 1 min. A volume of 1 mL of 1% aqueous sodium citrate (99%, Sigma-Aldrich Sydney, Australia) solution was then added and stirring continuously for 1 min. Following this, 1 mL of sodium borohydride (0.075 wt % dissolved in 1% aqueous sodium citrate) (99%, Sigma-Aldrich Sydney, Australia) was added and stirred for 5 min. The solution was stored at 4 °C away from light prior to further use.

Silicon was cut into 1 × 1 cm^2^ pieces and cleaned by immersing in a 1:3 (*v*/*v*) solution of hydrogen peroxide (30%, ChemSupply Gillman, Australia) and sulfuric acid (98%, RCI Labscan Bangkok, Thailand) at 80 °C for 15 min. The silicon was then removed, rinsed with water and dried with nitrogen. The silicon was then immersed in an 8.3 mM solution of 3-aminopropyltriethoxysilane (3-APTES) (99%, Sigma-Aldrich Sydney, Australia) in methanol (99.8%, Optigen Adelaide, Australia) for 24 h. The silicon pieces were then sonicated in methanol to remove any unbound silane, rinsed with methanol, and dried with nitrogen. A droplet of gold nanoparticle solution was then placed onto the silanized silicon surface and allowed to sit for 20 min, after which it was rinsed with water and dried gently with nitrogen.

### 2.3. AFM and SEM Calibration, and Operational Parameters

AFM imaging was performed using a Dimension FastScan system (Bruker Corporation, Billerica, MA, USA) with a Nanoscope V controller. The AFM samples imaged were a Nioprobe tip characterization sample (Aurora nanotechnology Nanaimo, BC, Canada), the above-mentioned gold nanoparticle sample, and a scanner calibration sample (Mikromasch model TGZ01: 3 µm pitch, 18 nm depth), which was used for ringing artefact investigation. AFM images were acquired in tapping mode and PeakForce tapping mode, with imaging parameters including set-point, scan rate, and feedback gains adjusted to optimize image quality and to minimize imaging force where possible. All images were flattened, and measurements of height and width were performed using the section tool of the Nanoscope Analysis software version 8.1 (Bruker Corporation, Billerica, MA, USA).

PeakForce tapping mode AFM engage parameters were set such that the force experienced by the CNT-AFM tip was minimized. The PeakForce Engage Set-point is the main parameter that must be controlled, and it was set at the minimum value to minimize contact force while avoiding false engagement. This parameter was initially set to 0.02 V (~1 nm), and then increased incrementally in 0.005 to 0.01 V steps until a successful engagement was obtained. The AFM scanner was calibrated in the x, y, and z directions using silicon calibration grids (Bruker Corporation, Billerica, MA, USA) (Bruker model numbers PG: 1 μm pitch, 110 nm depth, and VGRP: 10 μm pitch, 180 nm depth).

**Figure 2:**
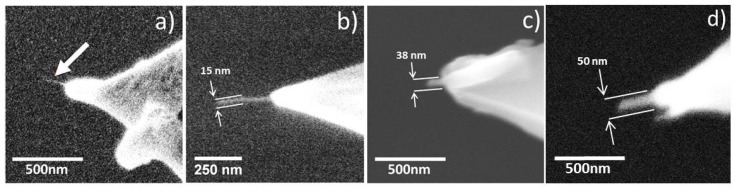
SEM images of the CNT probes used in this work, designated (**a**) CNT1. (**b**) CNT2. (**c**) CNT3 and (**d**) CNT4.

The AFM probes utilized in this work are as follows. For the tip seen in Figure 2a (CNT1), the probe model was Bruker OTR8 with nominal resonant frequency of 73 kHz, a nominal spring constant of 0.57 N/m and a nominal tip diameter of 15 nm. For the tip seen in Figure 2b (CNT2), the probe model was Bruker FastScan A with a nominal resonant frequency of 1400 kHz, a nominal spring constant of 18 N/m, and a nominal tip diameter of 10 nm. For the tip seen in Figure 2c (CNT3), the probe model was Bruker FMV with a nominal resonant frequency of 75 kHz, a nominal spring constant of 2.8 N/m, and a nominal tip diameter of 20 nm. For the tip seen in Figure 2d (CNT4), the probe model was Bruker ScanAsyst with a nominal resonant frequency of 70 kHz, a nominal spring constant of 4 N/m and a nominal tip diameter of 4 nm.

SEM measurements of CNT-AFM tips were acquired using a FEI Helios D433 Dualbeam SEM. The ion beam was not used for any imaging or cutting, so as to avoid damage to the delicate SWCNTs. The SEM was calibrated in the x and y dimensions, at the appropriate working distances and magnifications using the aforementioned calibration grids.

## 3. Results

CNT-AFM probes fabricated using the manual attachment method [38] were used in this study, and these are shown in Figure 2. CNTs were attached to AFM probes with nominal spring constants ranging from 0.57 to 18 Nm^−1^, and nominal resonant frequencies from 73 to 1400 kHz. In general, high resonant frequency is required for effective imaging in tapping mode (>100 kHz); PFT only requires a resonant frequency above the 0.5–8 kHz oscillation amplitude in order to avoid resonant effects.

**Figure 3:**
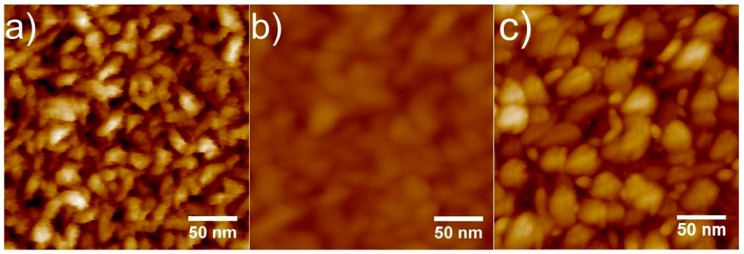
AFM images of the Nioprobe tip calibration sample in PeakForce tapping (PFT) mode using (**a**) probe CNT1 and (**b**) a new silicon FMV probe (nominal tip diameter of 20 nm) and (**c**) a new ScanAsyst probe (nominal diameter of 4 nm) The height or color scale for all images is 20 nm.

Application of the CNT-AFM probes for routine imaging was found to be very simple when using PFT mode. To demonstrate the imaging capability and the high resolution obtained, a Nioprobe tip calibration sample was imaged using CNT1 (Figure 2a), a standard new tapping mode probe (Bruker FMV type), and a new small diameter ScanAsyst AFM probe (Bruker ScanAsyst type). The resulting AFM image is shown in Figure 3. The sharp CNT-AFM probe provides superior resolution, clearly resolving the niobium grains present on the Nioprobe sample. The high aspect ratio of the CNT also allows for imaging deeper between the grains; as all images are displayed with the same height scale, the effect on image quality is dramatic. While the lateral resolution displayed in Figure 3b was not as good as that observed for the CNT-AFM probe, the ScanAsyst probe was able to provide better resolution than the FMV probe, and it could also image deeper between the grains when compared to the AFM image in Figure 3b. This is likely due to the smaller tip diameter and the higher aspect ratio for ScanAsyst probes compared to the FMV probes. Interestingly, even though the nominal tip diameter for the new ScanAsyst probe is quoted as 4 nm, the lateral resolution for the image was not as good as that for CNT1, which was determined to be approximately 5 nm (refer to Section 3.2). While the nominal diameter quoted by the manufacturer is 4 nm the maximum possible tip diameter quoted is 24 nm, indicating that tip diameters greater than 4 nm can occur for these types of probes. The other possibility that must be considered is that as the silicon probes become sharper, they also become more susceptible to wear, even when using such force-sensitive modes as PFT, particularly in air, where meniscus forces can induce relatively large imaging forces. Shearer et al. [6] noted this when imaging graphene layers with ScanAsyst tips in PFT mode.

### 3.1. Elimination of the “Ringing” Artefact

One of the most commonly encountered artefacts when imaging in tapping mode with a CNT-AFM probe is the “ringing” artefact described in detail by Park et al. [20] and Strus et al. [22]. Briefly, ringing artefacts are observed in tapping mode as a result of the adhesion of the laterally-flexible CNT to a steep, vertical feature on the surface. The adhesion of CNTs to surfaces can be very strong in many cases, and results in the oscillation of the probe being damped significantly. The result is a strong adhesion to and subsequent snap-off of the CNT from the surface, which is observed as a large high frequency oscillation or “ringing” at the edge of the feature where the adhesion occurs. This is then exacerbated by the gains used to track the surface, and makes imaging of these structures very difficult with CNT-AFM probes. This type of artefact has been observed on numerous samples, including, for example, DVD surfaces [40], silicon dots, the lines in an ArF resist pattern [20], and Tungsten nanorods [22].

A benefit of PFT is that feedback is based on the applied peak force, which is independent of adhesion during tip retraction; as a result, PFT should not suffer from the various adhesion-based artefacts observed with tapping mode. In order to test this and to further demonstrate the application of PFT to CNT-AFM probes, a calibration sample consisting of parallel trenches 20 nm deep (Mikromasch model TGZ01 Sofia, Bulgaria) was imaged in both tapping mode and PFT with the same CNT probe. The probe used was CNT2 shown in Figure 2b, a Bruker FastScan A type, with a nominal resonant frequency of 1400 kHz and a nominal spring constant of 18 Nm^−1^. While this is a relatively large spring constant for PFT, this cantilever type was chosen such that it was able to image effectively in both tapping and PFT modes.

A cantilever (type FMV) with a smaller spring constant was also tested, since although the FMV cantilever has a nominal resonant frequency of 75 kHz and a nominal spring constant of 2.8 Nm^−1^, they are still considered to be tapping mode probes, and therefore they could be used in tapping and PFT modes on the calibration sample. The CNT-AFM tip used in Figure 5 is CNT3 (as pictured in Figure 2c).

**Figure 4:**
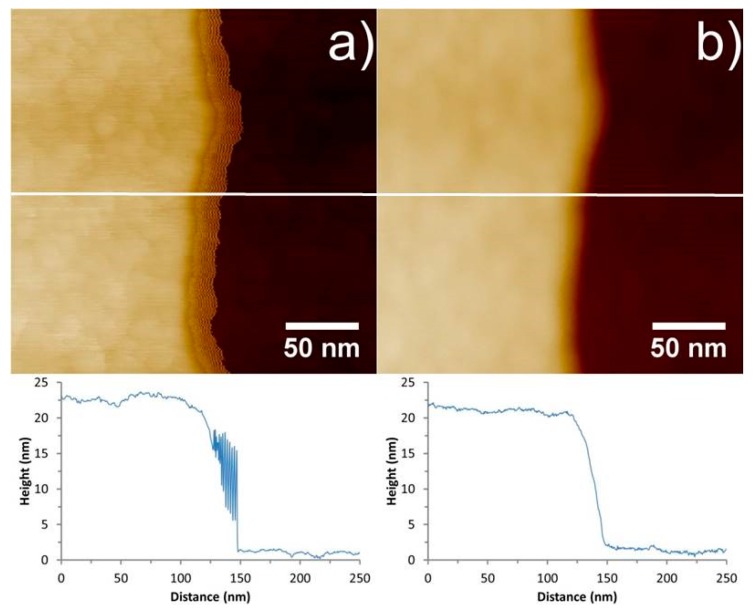
AFM images and corresponding cross sections of a Mikromasch TGZ01 calibration grid with 20 nm deep parallel trenches using the CNT2 probe. Image (**a**) was acquired in tapping mode, while image (**b**) was acquired using PFT. The white lines represent the positions of the cross sections underneath each image. From the images and cross sections it is obvious that the “ringing” artefact is eliminated when using CNT-AFM tips in PFT mode. The height or color scale for both images is 50 nm.

**Figure 5:**
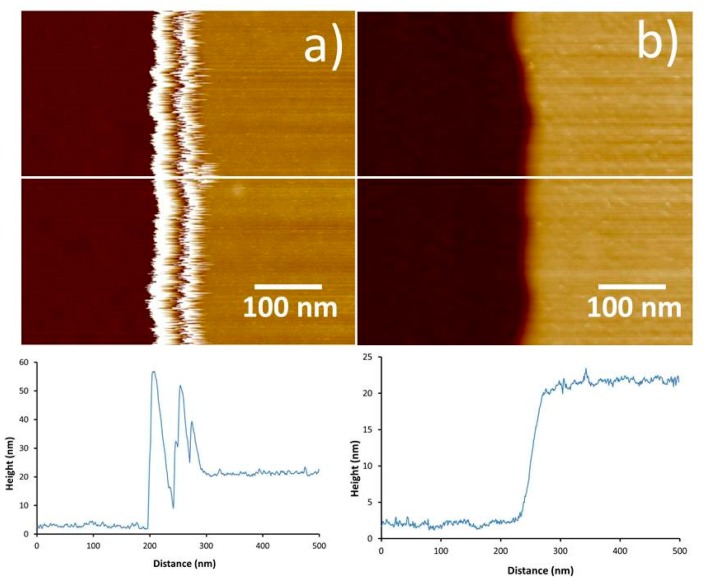
AFM images and corresponding cross sections of a Mikromasch TGZ01calibration grid with 20 nm deep parallel trenches using the CNT3 probe. (**a**) was acquired in tapping mode while (**b**) was acquired using PFT. The white lines represent the positions of the cross sections underneath each image. From the images and cross sections, it is obvious that the “ringing” artefact is eliminated when using CNT-AFM tips in PFT mode. The height or color scale for both images is 50 nm.

It is evident when comparing the tapping mode and PFT images for Figure 4 and Figure 5 that the “ringing” artefact for the CNT-AFM probes is eliminated in PFT mode. The benefits of PFT in the measurement of narrow trenches have been reported previously with standard probes [41]. Even when neglecting the geometry of a standard probe, in tapping mode, the amplitude is damped by adhesion with the trench walls, and it does not allow the probe to reach the base of the trench. The use of PeakForce tapping with CNT probes avoids the disadvantage of a low aspect ratio tip and neglects side-wall adhesion, providing an imaging mode that is well-suited to measuring deep, narrow trenches. This may prove to be an invaluable technique as the size of features in the semiconductor industry continue to decrease, and characterization becomes more and more challenging.

### 3.2. Determination of Thin CNT Diameters Using PFT Mode

**Figure 6:**
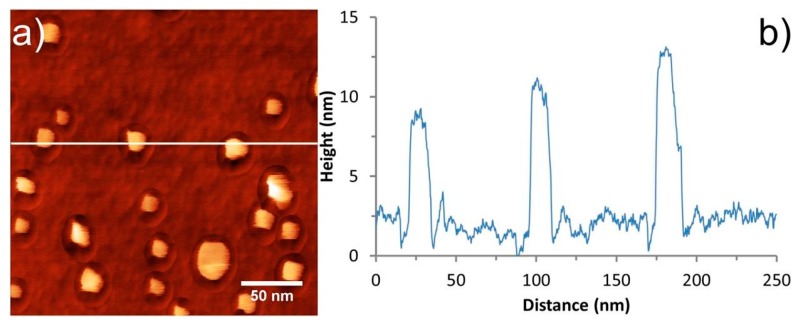
(**a**) AFM image of gold nanoparticles in PFT mode using probe CNT1, with (**b**) corresponding cross sections. The height or color scale for the image is 20 nm.

The diameter of the CNT attached to CNT-AFM probes, such as CNT2 and CNT3, for example, can be determined with reasonable accuracy using SEM imaging, as can be seen in Figure 2b,c. However a significant challenge when using very thin CNT-AFM probes, such as CNT1, is determining their diameters. A sample of gold nanoparticles (5–10 nm diameter) was used to characterize the diameter of the thinnest CNT probe CNT1 using images acquired in PFT mode. While not necessarily important for many imaging applications, for quantitative measurements, such as determining material properties, accurate knowledge of the tip or CNT diameter is crucial. There have been a number of reports of nanoparticles (often gold or polystyrene) being used to determine tip diameter and also calibrate piezoelectric scanners [42,43]. The cross-section of 10 gold nanoparticles from various images was used to determine the diameter of CNT1, with an image and cross section of the nanoparticles is shown in Figure 6. Using the approach taken by Vesenka et al. [44], Equation (1) was applied to the nanoparticle cross sections, where *W* and *h* are the measured width and height of the nanoparticle, respectively, and *R* is the tip’s radius of curvature. Equation 1 is determined from the contact geometry between two spheres representing the particle and the tip, as described by Vesenka et al. [44].

*W*^2^ = 8*Rh* (1)

The diameter of the CNT tip was determined, using Equation (1), to be 5.1 ± 0.7 nm, through the measurement of 10 different nanoparticles. However finding structures to characterize ultra-sharp CNT-AFM tips such as these is likely to be very challenging. Determining the diameter of the attached CNT for CNT1 using SEM is obviously difficult when referring to Figure 2a. The thermal motion of the CNT and the limits of the SEM resolution will result in a large degree of uncertainty.

### 3.3. Pit Artefact

**Figure 7:**
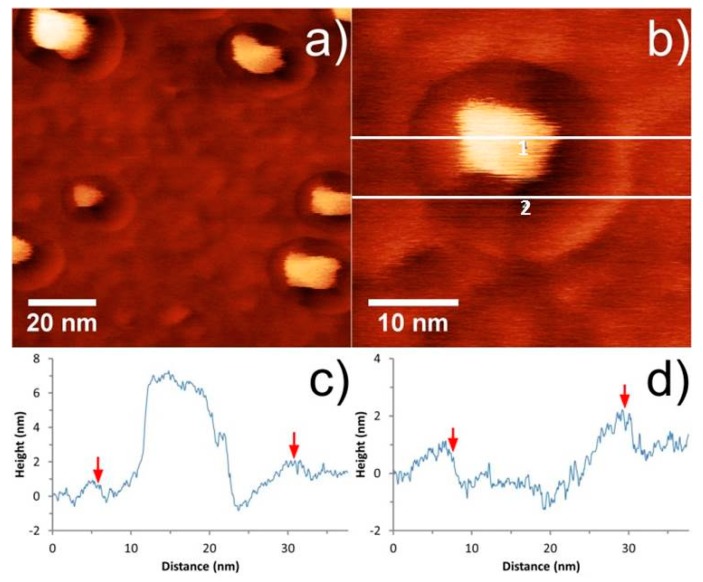
AFM images of gold nanoparticles on silicon using CNT1. (**a**) and (**b**) clearly showing the pit artefact with corresponding cross sections through the nanoparticle (line 1) and the pit (line 2). The edge of the pit, as observed by the AFM image, is indicated by the red markers in (**c**) and (**d**). The height or color scale for the image is 20 nm.

It was found that when imaging these small features with a sharp, thin CNT probe, an artefact resembling a “crater” or “pit” was observed around each of the nanoparticles. The pit was observed to extend a constant distance from the perimeter of the particle, independent of the particle size, and it varied in depth to some extent between the particles and around each individual particle. These features can be observed in Figure 6 and Figure 7, where the pit artefact is clearly visible around all of the nanoparticles in the images.

In order to explain the appearance of the pit, PFT feedback is briefly revisited, and the forces acting on a CNT tip during an oscillation cycle in close proximity to a nanoparticle are considered. The sample height in PFT mode is determined by the tip–sample distance at which the PeakForce set-point is reached. The tip (or sample) oscillates, bringing the tip and sample into intermittent contact, resulting in a peak repulsive force for each tap. The system tracks this peak force and adjusts the tip–sample separation to maintain the desired set-point.

**Figure 8:**
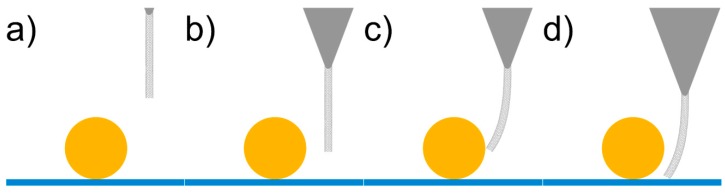
Schematic of a CNT tip during an oscillation cycle in the proximity of a nanoparticle, showing adhesion and buckling with the resulting height discrepancy.

Consider a CNT tip approaching the surface in the proximity of a nanoparticle, as shown in Figure 8. On approach, the CNT experiences attractive forces towards the nanoparticle. If the CNT is in close proximity, then it can make contact, while if the CNT is a greater distance away, then attractive forces may cause it to bend towards the nanoparticle before contacting the sample surface. In either case the CNT undergoes some degree of lateral bending before contacting the sample surface, which results in the effective length of the CNT decreasing. If the CNT effectively becomes slightly shorter in regions of high lateral adhesion, then the tip-sample separation must be decreased further to achieve the same cantilever deflection, thus resulting in the observation of a “pit” on the surface.

Meinander et al. reported an artefact of very similar origin for non-contact mode operation with CNT-AFM tips [45] when imaging platinum nanoparticles, and attributed this to the same adhesion effect.

**Figure 9:**
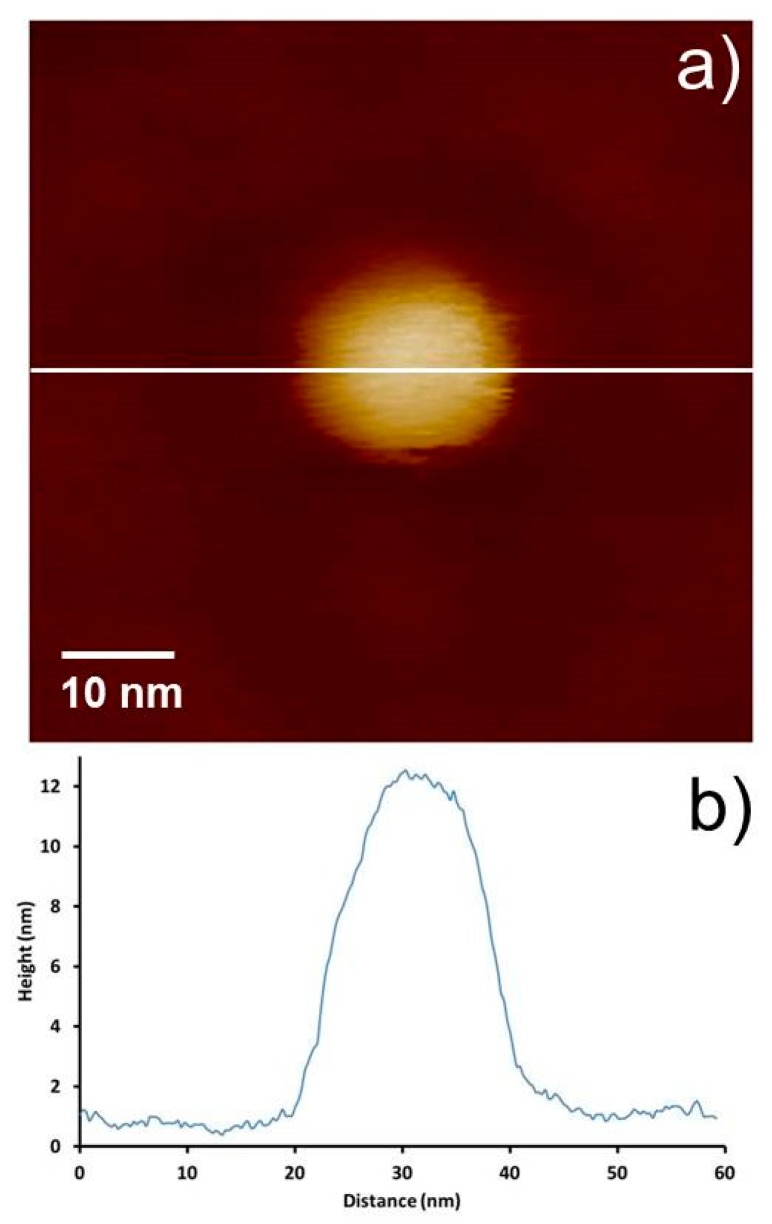
AFM image of a gold nanoparticle on silicon using CNT4. (**b**) shows the cross section that corresponds to the positon of the white line in (**a**). The “pit” artefact is significantly reduced due to the increased lateral stability of CNT4, due to the CNTs relative thickness. The height or color scale for the image is 20 nm.

Assuming that the degree of lateral bending of the CNT is dependent on its diameter and/or length, then this artefact should be reduced or eliminated for relatively short CNTs. This was tested by imaging the gold nanoparticle surface using a CNT-AFM probe with a CNT that was much thicker than CNT1. The CNT-AFM probe used was CNT4 from Figure 2 and the associated PFT AFM image can be seen in Figure 9. The cross section in Figure 9b reveals a significantly reduced “pit” artefact, and demonstrates that while relatively thick attached CNTs will not have the lateral resolution of thinner CNTs, the “pit” artefact can effectively be minimized in PFT mode using them, due to the increased lateral stability.

## 4. Discussion

The PeakForce tapping technique was investigated as an alternative mode for CNT-AFM probe imaging. The PFT mode was compared directly to the tapping mode by imaging vertical steps 20 nm in height. Tapping mode exhibited the adhesion-based “ringing” artefact, as expected, which was not present with images obtained by PFT. By considering only the peak interaction force for feedback, PFT is able to avoid certain artefacts caused by probe adhesion, which are incorporated into the feedback signal for tapping mode.

CNTs were attached to a low spring constant probe (CNT1), which is ideal for PFT imaging, and applied to gold nanoparticles as a method to determine the CNT diameter. Stable imaging was reported, and the gold nanoparticle sample provided a good resolution test and allowed the CNT diameter to be estimated as 5.1 *±* 0.7 nm. While very high resolution was observed, the nanoparticles were surrounded by a pit, which appears to be an artefact for CNT probes operating in PFT mode.

Attractive forces acting on thin CNTs are proposed to pull the CNT towards the nanoparticle when in close proximity. Lateral bending of the thin CNT will reduce the effective tip length, thus resulting in increased tip–sample separation and formation of a pit feature in close proximity to the nanoparticle. This demonstrates that while PFT mode is capable of eliminating artefacts that are associated with tapping mode, it is not completely immune from artefacts that are connected to surface forces when very thin CNT-AFM probes are used. However as long as the pit artefact is accounted for in any height and/or width analysis of structures on the sample surface, it will have a negligible effect, and it will also have a minimal effect on image resolution. We also show that the “pit” artifact can be minimized using a relatively thick CNT-AFM probe, albeit at the cost of lateral resolution. Further research with these types of AFM probes will focus on their applications in liquid, where CNT-AFM probes have proven to be difficult to apply, due to their hydrophobic nature [46,47]. Indeed some artefacts associated with CNT-AFM probes in air, such as the “ringing” artifact, could be reduced or eliminated, as capillary forces will also be reduced in liquid environments.
